# Outdoor school-based nature education programs improve social life and vitality: evidence from a six-wave longitudinal study on Italian secondary school students

**DOI:** 10.3389/fpsyt.2026.1913900

**Published:** 2026-09-23

**Authors:** Valeria Chiozza, Mei Xie, Susana Alves, Simona Grilli, Fabio Presaghi, Paola Perucchini, Marino Bonaiuto

**Affiliations:** 1 Department of Psychology of Developmental and Socialization Processes, Sapienza University of Rome, Rome, Italy; 2 Inter-University Research Centre in Environmental Psychology (CIRPA), Sapienza University of Rome, Rome, Italy; 3 Department of Human and Social Sciences, Universitas Mercatorum, Rome, Italy; 4 Department of Education, Roma Tre University, Rome, Italy

**Keywords:** civic engagement, contemporaneous crossed effects panel modeling, environmental education, longitudinal study, outdoor nature, secondary school students, social identity, vitality

## Abstract

**Introduction:**

This longitudinal study investigated how social identity predicts civic engagement and vitality over time among Italian secondary school students participating in school-based outdoor nature education activities.

**Methods:**

A total of 203 students participated in a six-wave longitudinal study conducted over one academic year (control group, *n* = 90; intervention group, *n* = 113). Three schools were involved, each implementing structured, school-specific outdoor nature education activities aimed at fostering psychological well-being, sense of belonging, and participation, including nature excursions, biodiversity projects, and outdoor group reflections. Longitudinal relationships among social identity, civic engagement, and vitality were examined using contemporaneous crossed-effects panel modeling in R.

**Results:**

Participation in the outdoor nature education program was associated with higher early levels of social identity and civic engagement compared with the control condition. Social identity significantly predicted civic engagement across multiple measurement waves, which in turn positively predicted vitality. A significant indirect effect of the intervention on vitality through social identity and civic engagement emerged during the initial intervention phase, whereas other indirect effects across subsequent waves were not statistically significant. The variability of effects over time may reflect developmental and contextual factors, including differences in program content and intensity across schools.

**Discussion:**

The findings support the role of social identity as a psychological foundation for civic engagement and suggest that school-based outdoor nature education may contribute to adolescents' psychological vitality by providing opportunities for belonging and meaningful collective participation. At the same time, variability across measurement waves and intervention contexts highlights the importance of considering program heterogeneity when evaluating the longitudinal effects of outdoor education initiatives.

## 1 Introduction

Adolescence represents a critical developmental period during which individuals undergo profound cognitive, emotional, and social transformations. At this stage, young people begin to develop more complex understandings of themselves and their place within the broader social world (1, 2). One of the key developmental tasks of adolescence is the formation of social identity—the sense of belonging to and identification with relevant social groups (3, 4). Social identities provide adolescents with psychological resources that shape how they interpret social information, make decisions, and engage with their communities (5). Importantly, these identities are not static; they evolve as adolescents navigate new peer groups, educational contexts, and societal structures.

A growing body of research highlights the role of social identity as a driving force for civic engagement (e.g., 6, 7). When adolescents identify strongly with a group—be it their school, neighborhood, nation, or peer networks, and the like—they are more likely to adopt group norms, internalize shared goals, and participate in activities that benefit the collective (8). This identification provides both motivational energy and a sense of responsibility toward collective issues, which can translate into various forms of civic engagement.

Civic engagement refers to attitudes and behaviors directed toward improving community and societal conditions, such as volunteering, participating in community projects, expressing opinions on social issues, or joining civic organizations (9, 10). Adolescence is an especially formative period for civic development because it is during these years that young people are exposed to civic ideas, encounter social diversity, and start to imagine their roles as active members of society (11). Early experiences with civic engagement can lay the groundwork for lifelong patterns of civic and political participation (12).

Beyond societal benefits, civic engagement is also closely linked to positive developmental outcomes at the individual level. Engaging in meaningful collective activities contributes to adolescents’ sense of agency, competence, and social connectedness, which are essential components of psychological well-being (13, 14). One particularly relevant indicator of well-being is subjective vitality, defined as the experience of feeling alive and energized (15). Vitality is not merely the absence of ill-being but reflects a positive, flourishing state that encompasses intrinsic motivation, purposeful activity, and sustained engagement with life (16). Empirical studies suggest that adolescents who are more civically engaged tend to report higher levels of vitality, self-esteem, and psychological resilience (17, 18).

However, much of the existing research on these relationships has been cross-sectional (19), limiting our understanding of how social identity, civic engagement, and vitality evolve dynamically over time. Longitudinal approaches are crucial for capturing the temporal sequencing of these constructs and identifying potential causal pathways of such a potential virtuous circle. For example, previous studies suggest that strong social identity may promote civic engagement, and engagement experiences can subsequently reinforce identity salience and adolescents’ sense of belonging (20). Likewise, vitality may not only result from civic engagement but can also act as a psychological resource that facilitates future participation (21). Examining these lagged associations allows researchers to move beyond static correlations and explore how earlier changes in one domain may shape later outcomes in another.

Moreover, relatively few studies have investigated these temporal processes within the context of secondary school–based outdoor education programs. Schools represent critical developmental settings where adolescents encounter structured opportunities to discuss societal issues, reflect on their identities, and participate in collective projects (22). A growing number of outdoor, school-based nature education initiatives aim to combine environmental learning with social and civic development. Well-designed outdoor programs can foster students’ sense of social belonging, provide meaningful participatory experiences, and ultimately enhance psychological well-being (23), over and above environmental attitudes and pro-environmental behaviors (24). Recent empirical research has provided increasingly consistent evidence that school-based outdoor and nature-based learning may support a broad range of developmental outcomes. A systematic review of 147 studies found that nature-specific outdoor learning was associated with socio-emotional, wellbeing, and academic benefits, with particularly relevant personal and social development outcomes among secondary-school students participating in adventure and outdoor education programs (25). More recently, a systematic review restricted to experimental and quasi-experimental school-led interventions found evidence of improvements in positive affect, physical activity, and social relationships or interactions among children and adolescents (26). Moreover, recent meta-analyses confirmed that school outdoor environmental education significantly improves both knowledge and attitudes regarding the environment among involved students (27). All these findings suggest that the benefits of nature-based education extend beyond environmental knowledge and academic learning to include psychological and interpersonal functioning. Nevertheless, the quality and design of the available studies remain heterogeneous, and relatively few investigations have employed repeated longitudinal measurements capable of clarifying the temporal relationships among social identification, participation, and wellbeing. Although repeated-measure studies have examined selected outcomes of school outdoor environments, such as physical activity (28), multi-wave evidence addressing psychosocial mechanisms remains limited.

The current study addresses these gaps through a six-wave longitudinal design involving secondary school students, some of whom participated in an intervention of outdoor education while others did not. By repeatedly measuring students’ social identity, civic engagement, and vitality over the course of a school year, we examine how these constructs are interrelated across time and how earlier changes predict later outcomes. The intervention condition consisted of a variety of school-specific outdoor nature education activities, which differed in content and intensity across schools. This naturalistic design allows us to examine developmental trajectories and potential causal effects. Specifically, we hypothesize that social identity at earlier time points will positively predict civic engagement at subsequent waves, which in turn will predict higher levels of vitality over time. We also expect that students in the intervention group will show stronger initial gains in social identity and civic engagement compared to the control group.

## 2 Conceptualizing school-based outdoor education in natural environments

Although the terms ‘outdoor education’, ‘nature-based education’ and ‘environmental education’ are frequently used interchangeably, they refer to related but distinct approaches. In the present study, outdoor education is considered the overarching framework, referring to educational practices that intentionally use outdoor environments as contexts for learning through direct and experiential engagement. Nature-based education more specifically focuses on learning about and through natural environments, whereas environmental education emphasizes the development of environmental awareness, values, and behaviors related to sustainability. Therefore, outdoor education represents the broader pedagogical approach, while nature- and environment-related components constitute specific dimensions of the educational experiences implemented.

A distinction is also made between “activities” and “programs”. The term “activities” refers to the specific learning experiences carried out within the intervention (e.g., outdoor tasks, experiential learning activities, and interactions with natural environments), whereas “program” refers to the structured and longitudinal intervention as a whole, consisting of multiple activities implemented across different time points during the school year. This distinction is particularly relevant in the context of the present longitudinal study, which examines changes over time associated with participation in an outdoor education program rather than the effects of isolated outdoor activities.

Within this framework, outdoor education is considered not only as a means to promote environmental learning, but also as a potential context for supporting adolescents’ social experiences, identity development, civic engagement, and psychological well-being.

## 3 Boosting Effects of Nature-Based Interventions on Adolescents

### 3.1 Social identity and civic engagement

Social identity theory (4) posits that individuals derive a sense of self from their membership in social groups, and this identification influences their attitudes, motivations, and behaviors toward collective goals. While it is often conceptualized in relation to proximal groups (e.g., school classes or peer networks), social identity can also extend to more inclusive and superordinate categories, such as identification with humanity or even the natural world (29). This broader form reflects a sense of belonging to larger social and ecological systems and may be particularly relevant in the context of environmental and nature-based experiences. In this vein, when adolescents strongly identify with meaningful groups—ranging from proximal contexts such as schools and peer networks to more inclusive categories such as communities or humanity—they internalize shared norms and values, which often include expectations about contributing to the public good (20). These identifications provide a crucial psychological foundation for civic engagement, shaping individuals’ sense of responsibility, efficacy, and motivation to act in ways that benefit their group and society at large (6).

This developmental linkage is especially relevant during early adolescence (approximately ages 12–14), when students are typically in lower secondary school (middle school). At this stage, young people undergo rapid cognitive, social, and emotional transformations, including the ability to adopt multiple social perspectives and reason about abstract collective concepts such as justice, fairness, and citizenship (1, 2). School becomes a particularly salient social context for identity development: peer networks expand, relationships with teachers become more complex, and students begin to see themselves as members of broader social communities. These experiences make early adolescence a sensitive period for the formation of social identities that can guide civic orientations and behaviors (30). Contemporary developmental research also emphasizes that civic engagement in early adolescence is embedded in relational and institutional contexts. Opportunities to participate, perceptions of being taken seriously, peer connectedness, and access to meaningful collective roles can influence whether adolescents translate civic attitudes into actual involvement (30, 31). School-based outdoor projects may provide these conditions by assigning students shared responsibilities, fostering collaborative problem-solving, and making the consequences of collective action tangible (8, 32).

Importantly, natural and outdoor settings can provide especially fertile contexts for the development of social identities and civic orientations during this stage (33). Shared experiences in nature—such as collaborative outdoor learning activities, field trips, or community-based environmental projects—create opportunities for adolescents to work toward common goals, build trust, and strengthen group bonds in ways that are often less constrained than traditional classroom settings (34, 35). Outdoor education programs frequently combine group-based challenges with meaningful engagement with the environment, thereby reinforcing both social connectedness and a sense of collective responsibility toward nature and the community (32). In addition, such experiences may foster more inclusive forms of social identification, extending beyond immediate peer groups to encompass broader communities, humanity, and the natural environment. For early adolescents, these experiences can play a formative role in shaping their sense of belonging to peer groups, schools, and local communities, laying the groundwork for sustained civic participation.

Recent evidence indicates that outdoor education may create conditions conducive to civic development when students are given opportunities to participate actively, collaborate with others, deliberate on shared problems, and contribute to visible environmental or community outcomes. In a systematic mixed-study review of 56 environmental education programs, Ardoin et al. (8) identified civic outcomes including increased participation, collective efficacy, community involvement, leadership, and action-oriented environmental engagement. The programs included in the review were diverse, but many lasted for more than one month, suggesting that civic outcomes are more likely to emerge when environmental learning is organized as a sustained participatory process rather than as a single exposure. Such findings are consistent with the view that shared environmental experiences may strengthen adolescents’ identification with social and ecological communities and provide concrete opportunities to enact this identification through collective action.

Empirical research consistently demonstrates a positive relationship between social identity and civic engagement. Adolescents who report higher levels of group identification are more likely to engage in volunteering, community participation, and political expression (7, 36). For example, Flanagan et al. (37) found that adolescents who felt connected to their school community were more likely to discuss political issues and participate in civic activities. Similarly, Sherrod et al. (11) showed that adolescents who develop a strong sense of social belonging during their school years exhibit greater civic involvement later in life.

In sum, social identity acts as a motivational driver and developmental scaffold, orienting young people toward civic goals and collective action. Strengthening not only proximal, but also inclusive forms of group identification during the middle school years—particularly through shared experiences in outdoor and natural contexts—may thus have long-term implications for civic engagement trajectories and psychological development into late adolescence and adulthood.

### 3.2 Social identity and vitality

Beyond its impact on civic behavior, social identity plays a crucial role in promoting psychological well-being during adolescence. According to Self-Determination Theory (14), the fulfillment of basic psychological needs—autonomy, competence, and relatedness—is fundamental to well-being and positive functioning. A sense of belonging to social groups—whether proximal or more inclusive—satisfies the need for relatedness and provides adolescents with a stable framework for meaning-making, emotional support, and social validation (2, 3).

The social cure framework (38) further argues that social identities protect and enhance health by providing access to social support, collective efficacy, and a sense of control. Adolescents with strong social identities typically experience lower stress, more effective coping strategies, and higher levels of psychological well-being (6, 39). These benefits may also extend to broader forms of identification, as feeling connected to humanity or to the natural world can provide meaning, continuity, and a sense of shared purpose beyond immediate social contexts (40). This is especially significant during early adolescence (approximately ages 12–14)—a period that often corresponds to the transition into lower secondary school—when young people face new academic, social, and emotional demands. At this stage, peer relationships become increasingly central, while the need for belonging intensifies as students navigate larger and more complex school environments. A strong sense of group identification during this developmental window can provide essential psychological resources that buffer stress and foster adaptive adjustment (41).

Importantly, contact with nature and participation in outdoor educational activities can further amplify these identity-related benefits for psychological well-being. In terms of Social Determination Theory, natural environments offer unique opportunities for fulfilling basic psychological needs: they support autonomy through unstructured exploration, competence through hands-on challenges, and relatedness through shared experiences with peers (34, 35). In terms of Social Identity Theory, they may also encourage adolescents to develop a more expansive sense of identity, fostering feelings of connectedness not only with peers but also with broader ecological systems (42). Moreover, the Attention Restoration Theory (43) suggests that exposure to nature restores depleted cognitive and emotional resources, helping adolescents feel more energized and focused. Similarly, Ryan et al. (44) argue that nature contact promotes intrinsic motivation and vitality by fostering feelings of connectedness and authenticity. Recent intervention studies and systematic reviews support the relevance of these mechanisms in school settings. Ly and Vella-Brodrick’s (2024) (26) review of experimental and quasi-experimental school-led nature interventions found evidence of benefits particularly for positive affect and social interactions, although effects varied according to the characteristics of the intervention and the participants. In an Italian study involving primary- and secondary-school students, participation in outdoor environmental education was associated with improved psychophysical wellbeing, connectedness to nature, and prosociality compared with a control condition (23). These findings are particularly relevant to subjective vitality because they indicate that direct and socially organized contact with nature can simultaneously provide restorative experiences, positive emotional activation, and opportunities for interpersonal connection.

Further evidence comes from a recent school-based intervention involving 211 Italian high-school students. The MINDhEARTH program, which combined nature-related and mindfulness-based activities, produced improvements in subjective happiness, autonomy, and selected mindfulness dimensions, although changes in measured nature connectedness were not statistically significant (45). The mixed pattern of results is important because it suggests that nature-based programs do not uniformly affect all psychological outcomes and that their effects may depend on program content, intensity, implementation, and the specific developmental processes targeted.

For early adolescents, outdoor experiences such as group hikes, environmental projects, or field-based learning can provide powerful opportunities to consolidate social identities while simultaneously enhancing psychological energy and well-being (46).

Vitality, defined as the subjective experience of feeling alive, energetic, and psychologically vigorous (15), represents a core component of adolescent flourishing. Belonging to valued social groups can directly enhance vitality by providing meaningful goals, emotional connection, and motivation to participate in life activities (44). For early adolescents, who are simultaneously consolidating their identities and expanding their social worlds, group belonging can be particularly energizing. Shared goals and supportive peer networks help channel adolescents’ emerging agency into purposeful action, promoting a sense of enthusiasm and engagement with their surroundings (47).

Empirical studies have shown that adolescents with stronger social identities report higher levels of vitality and lower levels of depressive symptoms, largely due to increased feelings of support and shared purpose (39, 48). In outdoor education contexts, collective experiences in natural settings can strengthen these identity-based resources, providing both immediate affective benefits and longer-term developmental gains. Group activities and social identity, especially coupled with outdoor natural settings, thus serves not only as a protective factor against stress and maladjustment but also as a positive developmental asset that contributes to adolescents’ motivation, resilience, and subjective well-being (49). In short, social identity —particularly when it encompasses broader and more inclusive forms of belonging—shapes not only adolescents’ external civic behaviors but also enriches their internal psychological states, fostering thriving and flourishing during this pivotal life stage. This later effect is synergistic with traditional beneficial cognitive and affective effects of experiencing natural places (50).

### 3.3 The mediating role of civic engagement

Civic engagement serves as a key developmental bridge between social identity and psychological well-being. When adolescents identify strongly with their communities, they are more likely to engage in civic activities that reflect their group’s norms and values (51). Such activities—including volunteering, participating in environmental initiatives, or contributing to local community projects—offer opportunities for competence development, social connection, and autonomy, which are crucial for well-being and vitality (13, 14).

Outdoor education offers a particularly powerful experiential context for these processes to unfold. Defined broadly, outdoor education involves structured learning experiences that take place outside traditional classroom settings, often emphasizing collaborative learning, real-world problem solving, and meaningful interaction with the environment (34, 35). These activities typically integrate cognitive, social, and emotional dimensions of learning, making them especially suited to the developmental needs of adolescents. Outdoor settings also provide more flexible and less hierarchical environments that can empower students who may struggle to express agency or leadership within rigid classroom structures (52).

The benefits of outdoor education are especially salient during early adolescence (approximately ages 12–14), when students are typically enrolled in lower secondary school. This is a period characterized by growing social awareness, identity exploration, and heightened sensitivity to peer relationships (1, 2). Early adolescents are actively negotiating their sense of self within expanding social networks while seeking meaningful roles beyond their immediate family context. Outdoor environments support these developmental tasks by encouraging autonomy, peer collaboration, and experiential learning.

Within this framework, outdoor education and nature-based contexts provide ideal platforms for civic engagement to emerge as a mediating mechanism between identity and well-being. Activities such as environmental restoration projects, biodiversity monitoring, tree planting, community gardening, or guided nature walks enable adolescents to contribute tangibly to their communities while simultaneously fostering environmental stewardship (53). These hands-on experiences link adolescents’ emerging social identities with meaningful collective action in ways that are often more immediate and emotionally engaging than classroom-based learning. Working together toward a shared ecological goal can deepen adolescents’ sense of belonging and reinforce civic values related to sustainability, cooperation, and responsibility toward the natural world (34, 35).

Evidence from the environmental education and positive youth development literature suggests that the developmental value of these experiences depends not only on exposure to nature but also on the role assigned to young participants. Programs that involve decision-making, shared responsibility, problem solving, cooperation, and contributions to community goals are more likely to support agency, competence, belonging, and civic development. A systematic review of environmental education outcomes relevant to positive youth development identified recurring benefits in domains such as confidence, competence, connection, character, and contribution, while also highlighting considerable variation in research designs and outcome measurement. This literature supports the interpretation of civic engagement as an active developmental mechanism: students do not benefit solely because they are outdoors, but because outdoor and environmental experiences allow them to participate meaningfully, cooperate with peers, and observe the consequences of their actions.

Recent qualitative evidence from outdoor adventure education further shows that adolescents and educators perceive social connection, tangible responsibilities, manageable challenges, and experiences of success as central to the psychosocial benefits of outdoor programs. These program characteristics may enhance empowerment and collective efficacy and are therefore consistent with the proposed pathway from inclusive social identity to civic engagement and, subsequently, psychological vitality (54).

Civic engagement during this developmental stage is especially potent. Early adolescence marks the transition from primary to lower secondary school, a period in which cognitive and moral reasoning abilities expand, allowing young people to think more abstractly about collective issues such as fairness, environmental protection, and social justice (1). Outdoor civic experiences during this time can be developmentally catalytic, offering adolescents meaningful opportunities to enact their identities, collaborate with peers, and contribute to causes larger than themselves.

Several empirical studies support this mediating pathway. Bliuc et al. (36) found that group identification predicted political participation, which in turn was associated with increased psychological empowerment. Ballard et al. (17) demonstrated that civic engagement contributes to adolescents’ sense of purpose and agency, both of which are important predictors of well-being. Crocetti et al. (20) showed that civic participation strengthens identity salience over time, creating a positive feedback loop between identity, engagement, and psychological outcomes.

Engaging in civic activities gives adolescents the chance to enact their identities, receive feedback from peers and communities, and experience efficacy in meaningful domains. For early adolescents in particular, outdoor and nature-based civic activities make these developmental processes especially salient by combining hands-on action, peer collaboration, and tangible community impact (55). These structured opportunities help channel their growing social awareness into purposeful action, supporting both identity consolidation and personal growth. Civic engagement thus functions not only as a behavioral manifestation of social identity but also as a psychological mechanism that promotes flourishing, mediating the link between identity and well-being across adolescence. A classic example of such a virtuous chain is the century old scout movement, globally spread, which joins group and social identity formation, civic engagement, and broader well-being and social adaptation.

In sum, in the Italian secondary school context, outdoor education provides an innovative and contextually relevant platform for interventions aiming to foster civic identity and engagement. By integrating social identity development, community participation, and psychological well-being within a single experiential framework, outdoor education serves as a naturalistic and developmentally appropriate setting for promoting long-term civic and personal outcomes—particularly during the formative early adolescent years.

## 4 Methods

### 4.1 Participants and procedure

Based on the path model shown in **Figure 1**, the optimal sample size, given a small-to-medium direct effect (b = 0.30) and a small mediation effect (b = 0.09, b = 0.027), was computed using the Monte Carlo method developed by Wang and Rhemtulla (2021) (56). Assuming an average auto-regressive effect of b = .50, a direct effect of the Treatment on the mediations of b = .30, a direct effect of the mediator on the other mediator or the main outcome of b = .30, and a small direct effect of the Treatment on the main outcome of b = .10, we obtained that with a sample of N, 200 participants, the power for detecting the effect of the Treatment on mediators (setting critical alpha at 0.05, and running 1000 Monte Carlo simulations) is about 1 − β = .996. Additionally, the direct effect of the mediator on another mediator or on the outcome, the expected average power is approximately 1 − β = 0.983. Finally, on average, the power to detect a small indirect effect (ranging from b = 0.027 to b = 0.09) is approximately 1 − β = 0.932. Considering also an average impact of 25% of missing values shrinking the sample size to N, 150, the expected average power for direct Treatment effect on mediators is of about 1 − β = .984; while the average direct effect of mediator on the other mediator or the outcome is about 1 − β = .955; and the average power for indirect effects is about 1 − β = .823.

**Figure 1:**
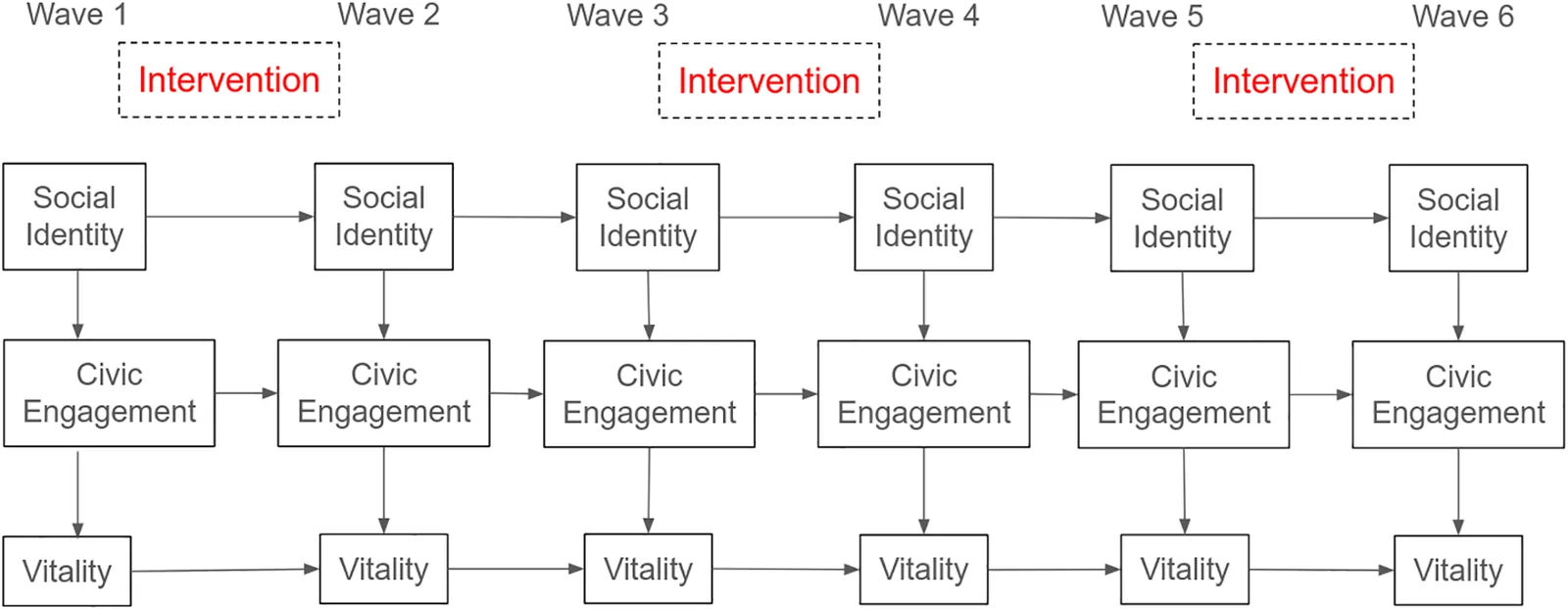
The proposed model. The interventions were administered at Waves 1, 3, and 5, and their paths are not shown in the path model but are included in the path analysis.

Participants were 203 Italian secondary school students (52% girls; aged 13–17 years) recruited from three Italian schools. A longitudinal cluster randomized trial was conducted with two conditions (intervention vs. control) and six measurement waves spanning one academic year. Classes were assigned by teachers either to the intervention group (*n*, 113) or the control group (*n*, 90). The intervention group participated in an outdoor nature education program that combined environmental learning with experiential activities in natural settings. Because the sample included multiple schools, specific activities varied slightly across institutions, reflecting differences in local environmental resources and community partnerships. Across three intervention phases (T1–T2, T3–T4 and T5–T6), each participating school organized three sets of thematic workshops and excursions designed to foster students’ sense of belonging, environmental responsibility, and connection with nature. The participating schools were located in different regions of Italy, namely Frosinone (Lazio, central Italy), Reggio Calabria (Calabria, southern Italy), and Valderice (Sicily, southern Italy), thereby providing diverse environmental and socio-cultural contexts. Activities varied accordingly across sites: for example, students in Reggio Calabria engaged in guided visits to natural environments (e.g., beaches), those in Sicily participated in nature hikes (e.g., in the Monte Cofano Nature Reserve), and those in Frosinone carried out biodiversity projects in school gardens and courtyards. Additional activities included botanical experiments inspired by Mendelian genetics, outdoor reading sessions, and group reflections (see **Figure 2** for examples). These experiences were intentionally structured to integrate identity-building (e.g., shared group experiences in natural and cultural settings), environmental engagement (e.g., stewardship activities, collaborative outdoor projects), and psychological vitality promotion (e.g., immersion in nature, hands-on exploration). Each intervention phase was coordinated by the participating schools in collaboration with the research team and implemented during the corresponding period of the academic year. Outdoor activities were conducted by classroom teachers in collaboration with local environmental educators or trained facilitators when available, depending on the specific school. Researchers monitored the implementation of the outdoor nature education program throughout the academic year through regular communication with participating schools and the collection of documentation describing the activities carried out. Although the specific content varied across schools to reflect local environmental resources, all interventions shared the common objectives of promoting environmental learning, social participation, and psychological well-being.

**Figure 2:**
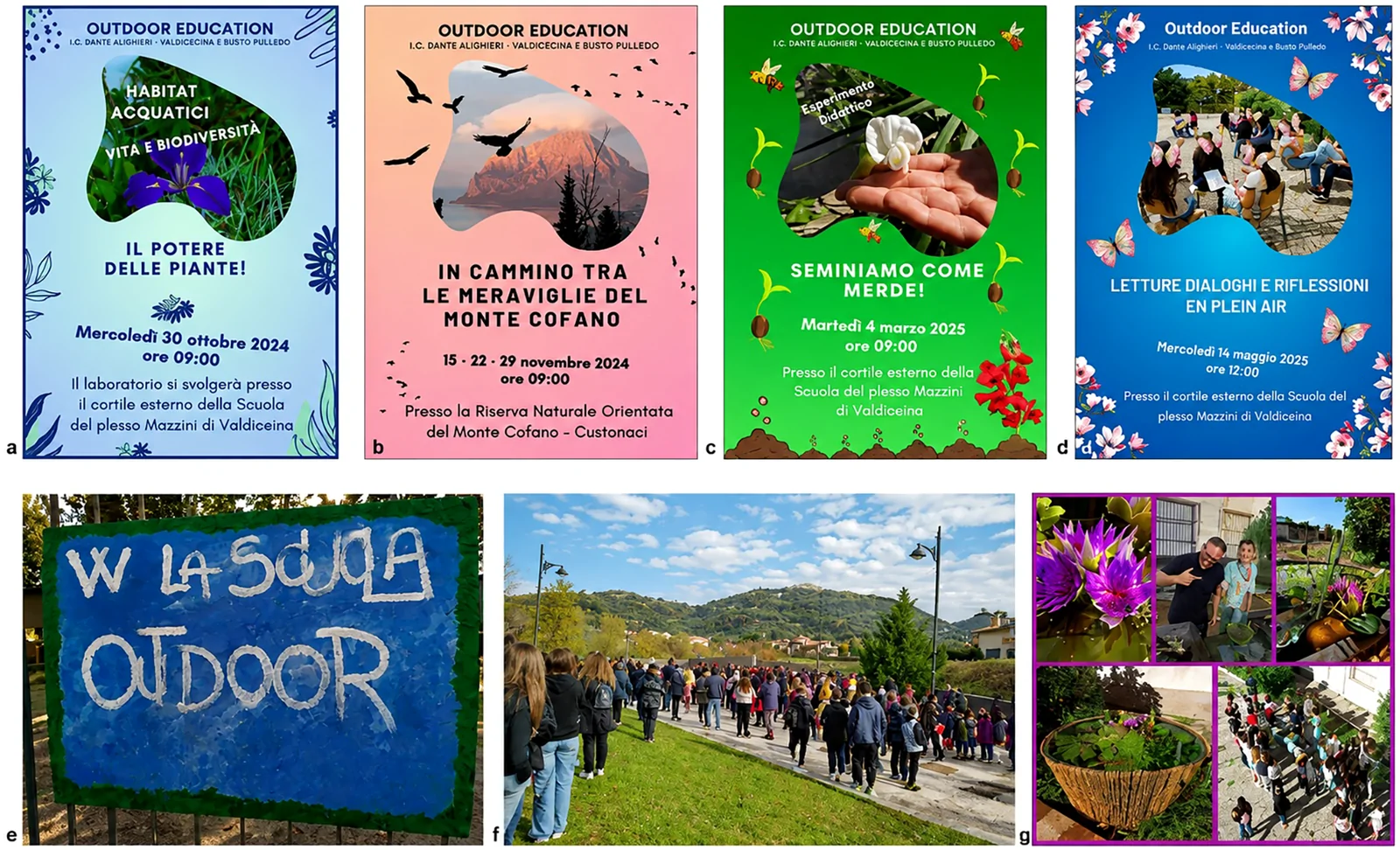
Environmental outdoor education examples. Examples of outdoor education activities implemented across the three intervention phases. **(a–d)** display promotional materials for the different activities conducted in participating schools: **(a)** aquatic habitats workshop (*“Il potere delle piante”*), **(b)** nature excursion in the Monte Cofano Nature Reserve, **(c)** botanical laboratory inspired by Mendelian genetics, and **(d)** outdoor reading and group reflection sessions. **(e–g)** show photographs from the activities, including outdoor learning settings, group excursions, and biodiversity projects carried out in school gardens and natural environments. Activities were organized autonomously by each school and varied according to local environmental contexts and educational approaches.

In contrast, students in the control group followed the standard science curriculum, which included classroom-based instruction on environmental and sustainability topics. This instruction primarily focused on the acquisition of factual knowledge and conceptual understanding. However, it did not include structured outdoor, group-based experiential activities, nor did it explicitly target identity-related processes, environmental engagement through practice, or psychological vitality through direct contact with natural environments. While both groups were exposed to similar thematic content related to environmental issues, the intervention group additionally engaged in nature-based, collaborative activities specifically designed to foster students’ sense of belonging, environmental responsibility, and connection with nature through experiential learning. These activities integrated identity-building processes, environmental stewardship actions, and immersive experiences in natural and cultural settings. This distinction allows us to isolate the added value of experiential, group-based outdoor engagement beyond classroom-based knowledge transmission alone.

Baseline demographic information included participants’ age and gender. No direct measures of socioeconomic status, family background, physical or mental health status, or ethnicity were collected, as the primary objective of the project was to evaluate changes in social-psychological outcomes associated with participation in school-based outdoor nature education activities.

The study comprised six measurement points: Pre-test (T1), Post-Intervention 1 (T2), Pre-Intervention 2 (T3), Post-Intervention 2 (T4), Pre-Intervention 3 (T5), and Post-Intervention 3 (T6). The three rounds of intervention activities were conducted between T1–T2, T3–T4, and T5–T6. This multi-wave design enabled the longitudinal assessment of developmental changes and intervention effects on social identity, civic engagement, and vitality over time.

Longitudinal invariance of latent constructs (Social Identity, Civic Engagement, and Vitality) was evaluated in the pooled sample.

### 4.2 Measures

All scales were translated and back-translated between Italian and English to ensure both linguistic and conceptual equivalence.

#### 4.2.1 Social identity

Social identity was measured at each wave using the *Global Identity Scale* (57), which captures identification with a superordinate social category, namely humanity and the natural world. Participants rated their identification with the world and its people on three items: *“I see myself as a global citizen”, “I feel connected with the whole earth”*, and *“I feel as a part of the earth”.* Minor adaptations to the original wording were introduced to better fit the Italian language and the age of the sample. Responses were provided on a 7-point Likert scale (1, strongly disagree; 7, strongly agree). Internal consistency coefficients were acceptable across waves (Cronbach’s α: T1 = .706; T2 = .784; T3 = .839; T4 = .866; T5 = .860; T6 = .846).

#### 4.2.2 Civic engagement

Civic engagement was assessed with a six-item scale developed by Doolittle and Faul (58), rated on a 5-point Likert scale (1, strongly disagree; 5, strongly agree). To better fit the school context, the word “community” was replaced with “school community”. Example items include: *“I believe I should make a difference in my school community”, “I am committed to serve in my school community”*, and *“I believe that all citizens have a responsibility to their community”.* Cronbach’s α indicated excellent internal consistency at all time-points (T1 = .763; T2 = .812; T3 = .794; T4 = .864; T5 = .875; T6 = .883).

#### 4.2.3 Vitality

Subjective vitality was measured with the six-item *Subjective Vitality Scale* (15), which captures students’ subjective sense of energy and aliveness. Participants rated each item on a 7-point Likert scale (1, not at all true; 4, somewhat true; 7, very true). Example items included: *“I feel alive and vital”* and *“I have energy and spirit”*. Internal consistency was excellent across all six waves (Cronbach’s α: T1 = .684; T2 = .627; T3 = .641; T4 = .741; T5 = .714; T6 = .822).

Overall, internal consistencies were satisfactory across all measures and time points (Cronbach’s α >.60).

### 4.3 Data analysis

Missing data were handled using full information maximum likelihood (FIML) estimation, which provides unbiased parameter estimates under the assumption of missing at random (MAR; 59). Preliminary analyses indicated that data were missing at random (MAR), as Little’s MCAR test was non-significant (*p* >.05).

Before estimating the longitudinal structural model, we evaluated longitudinal measurement invariance for social identity, civic engagement, and vitality across the six measurement waves. A hierarchical sequence of longitudinal confirmatory factor models was estimated separately for each construct. First, configural invariance was tested by specifying the same factor structure across waves with no constraints on any estimated parameter. Second, longitudinal metric invariance was examined by constraining corresponding factor loadings to equality over time. Third, longitudinal scalar invariance was tested by additionally constraining the corresponding item intercepts to equality across waves. Finally, strict invariance was evaluated by also constraining item residual variances to equality over time. To limit model complexity in the six-wave design, civic engagement and vitality were assessed using item parcels, while social identity was modeled using its three individual indicators.

In evaluating model fit, we considered not only the chi-square test but also the Root Mean Squared Error of Approximation (RMSEA) and its 90% confidence interval, adopting values of.08 or lower as indicating acceptable fit, as well as the Comparative Fit Index (CFI) and the Tucker-Lewis Index (TLI), for which values of.90 or higher were considered acceptable (60). To assess whether a specific measurement invariance is achieved, a series of hierarchical statistical tests was performed by comparing the chi-squared value of the more complex model (i.e., the configural model) with that of the less complex model (i.e., the model with invariant factor loadings). If the difference is not statistically significant, we concluded that measurement invariance was achieved and proceeded to the next comparison. The hierarchical tests were performed until a non-significant test was found.

Regarding the model fitted for testing our research hypothesis (**Figure 1**), we fitted a contemporaneous crossed effects panel model (61–63) using the lavaan package (v0.6–19) in R. The group variable (R) was dummy-coded (0, control, 1, intervention) and included as an exogenous predictor in all models. The model included autoregressive and contemporary effects across constructs, as well as group assignment (R) as an exogenous predictor. Finally, since the intervention was repeated three times, we covaried the residuals at T1, T3, and T5 to account for the common method factor. Indirect effects were assessed using Monte Carlo 95% confidence intervals (50,000 replications). All models were estimated using robust maximum likelihood (MLR) to account for non-normality in the Likert-type indicators. Because participants were nested within three schools, the clustering effect was evaluated. Given the small number of clusters (k, 3), multilevel modeling was not feasible; therefore, analyses were conducted at the individual level, and the potential impact of school-level clustering is considered in the limitations section. As a supplementary analysis, we examined whether the observed mean trajectories of social identity, civic engagement, and vitality differed according to gender. Three heteroscedasticity-robust repeated-measures ANOVAs were conducted, one for each observed scale score. Time, comprising the six measurement waves from T1 to T6, was specified as the within-subject factor, and gender was specified as the between-subjects factor. A White heteroscedasticity-consistent adjustment was applied. Participants who selected non-binary/third gender or preferred not to report gender were excluded from the supplementary gender analyses because the number of cases in these categories was insufficient for meaningful between-group comparisons. Gender-related differences were examined in a supplementary sensitivity analysis. The results of these preliminary analyses were considered before interpreting the longitudinal structural paths.

## 5 Results

### 5.1 Attrition

To assess whether dropout at different waves was associated with differences in baseline measures, a series of ANOVAs were conducted with the Social Identity, Civic Engagement, and Vitality at T1 as the dependent variables and dropout status (completer vs drop-out) at each successive wave (T2, T3, T4, T5, and T6) as the independent variable. Results indicated no statistically significant differences for Social Identity or Civic Engagement across any wave. For Vitality, a significant difference emerged only for dropout at T2, *F*(1, 127) = 5.95, *p* = .016, whereas no other comparisons reached statistical significance. Overall, these findings suggest that attrition was largely unrelated to baseline characteristics, with limited evidence of selective dropout (see **Supplementary Table S1** in **Supplementary File 1**).

### 5.2 Preliminary analyses

#### 5.2.1 Longitudinal measurement invariance

Results of the longitudinal measurement invariance analyses are reported in **Tables 1**–**3**. For social identity, configural, metric, and scalar invariance were supported, whereas strict invariance was not supported (**Table 1**).

**Table 1:** Longitudinal measurement invariance for social identity.

Model									RMSEA 90% C.I.				
	Df	AIC	BIC	χ^2^	Δχ^2^	Δdf	P	RMSEA	LL	UL	CFI	TLI	Δ-CFI
Configural Invariance	75	5266.7	5649.3	121.38				.054	.036	.071	.959	.917	--
Weak Invariance	90	5256.0	5588.3	140.71	19.34	15	.199	.052	.034	.067	.955	.924	.004
Scalar Invariance	105	5244.9	5526.8	159.57	18.86	15	.220	.050	.033	.065	.952	.930	.003
Strict Invariance	120	5257.4	5489.0	202.07	42.51	15	.0001	.057	.043	.070	.928	.908	.024

Df, Degree of Freedom; AIC, Akaike Information Criterion; BIC, Bayesian Information Criterion; χ2, Chi-squared test value; Δχ2, Differential Chi-squared test value between two nested models; Δdf, differential degree of freedom; P, p-value; RMSEA, Root Mean Squared Error of Approximation; LL, 90% C.I. Lower Limit; UL, 90% C.I. Upper Limit; CFI, Comparative Fit Index; TLI Tucker-Lewis Index; Δ-CFI, Differential CFI between two nested models.

**Table 2:** Longitudinal measurement invariance for civic engagement.

Model									RMSEA 90% C.I.				
	Df	AIC	BIC	χ^2^	Δχ^2^	Δdf	P	RMSEA	LL	UL	CFI	TLI	Δ-CFI
Configural Invariance	75	7921.4	8304.0	110.96				.048	.027	.066	.960	.919	--
Weak Invariance	90	7909.2	8241.5	128.76	17.80	15	.273	.045	.026	.062	.957	.927	.003
Scalar Invariance	105	7895.0	8177.0	144.61	15.85	15	.392	.042	.023	.058	.956	.936	.001
Strict Invariance	120	7893.9	8125.5	173.49	28.88	15	.017	.046	.030	.060	.941	.924	.015

Df, Degree of Freedom; AIC, Akaike Information Criterion; BIC, Bayesian Information Criterion; χ2, Chi-squared test value; Δχ2, Differential Chi-squared test value between two nested models; Δdf, differential degree of freedom; P, p-value; RMSEA, Root Mean Squared Error of Approximation; LL, 90% C.I. Lower Limit; UL, 90% C.I. Upper Limit; CFI, Comparative Fit Index; TLI Tucker-Lewis Index; Δ-CFI, Differential CFI between two nested models.

**Table 3:** Longitudinal measurement invariance for vitality.

Model									RMSEA 90% C.I.				
	Df	AIC	BIC	χ^2^	Δχ^2^	Δdf	P	RMSEA	LL	UL	CFI	TLI	Δ-CFI
Configural Invariance	75	10489	10871	128.11				.058	.040	.075	.971	.941	
Weak Invariance	90	10482	10815	151.78	23.67	15	.071	.057	.041	.072	.966	.943	.005
Scalar Invariance	105	10473	10755	172.22	20.44	15	.156	.055	.040	.069	.963	.947	.003
Strict Invariance	120	10467	10698	196.24	24.02	15	.056	.055	.041	.068	.958	.947	.005

Df, Degree of Freedom; AIC, Akaike Information Criterion; BIC, Bayesian Information Criterion; χ2, Chi-squared test value; Δχ2, Differential Chi-squared test value between two nested models; Δdf, differential degree of freedom; P, p-value; RMSEA, Root Mean Squared Error of Approximation; LL, 90% C.I. Lower Limit; UL, 90% C.I. Upper Limit; CFI, Comparative Fit Index; TLI Tucker-Lewis Index; Δ-CFI, Differential CFI between two nested models.

Civic engagement showed the same pattern, achieving configural, metric, and scalar invariance but not strict invariance (**Table 2**).

By contrast, vitality achieved strict longitudinal measurement invariance (**Table 3**).

Overall, all three constructs met at least the scalar invariance criterion, supporting the longitudinal comparison of latent means and structural associations across the six measurement waves. The lack of strict invariance for social identity and civic engagement indicates that indicator-specific residual variances were not fully stable over time; however, it does not, by itself, imply that the substantive meaning of these constructs changed across measurement occasions.

#### 5.2.2 Gender-related differences over time

As a supplementary robustness analysis, gender differences in the trajectories of social identity, civic engagement, and vitality across the six measurement waves were examined using repeated-measures ANOVAs.

Descriptive statistics indicate that boys generally reported slightly higher mean scores than girls across several measurement occasions, particularly for civic engagement and vitality (**Table 4**).

**Table 4:** Means and standard errors for Social Identity, civic engagement, and vitality by gender across the six measurement waves.

Time	Social identity				Civic engagement				Vitality			
	Boys		Girls		Boys		Girls		Boys		Girls	
	M	SE	M	SE	M	SE	M	SE	M	SE	M	SE
T1	13.44	0.60	12.39	0.38	23.75	1.39	21.41	0.95	31.33	2.19	26.04	1.37
T2	13.67	0.70	12.74	0.44	27.13	1.30	23.76	0.89	30.78	2.14	29.22	1.34
T3	12.56	0.82	12.52	0.51	25.25	1.30	24.29	0.89	33.78	2.29	29.26	1.43
T4	14.56	0.70	12.74	0.44	26.50	1.13	24.29	0.77	32.56	2.14	30.00	1.34
T5	13.67	0.57	13.04	0.35	25.00	1.20	24.06	0.83	31.00	2.20	30.61	1.38
T6	13.78	0.65	12.57	0.41	28.00	1.41	25.06	0.97	33.56	1.80	30.35	1.12

The repeated-measures ANOVAs revealed no statistically significant main effects of gender, no significant main effects of time, and no significant Gender × Time interactions for any of the three constructs (**Table 5**).

**Table 5:** Results of the repeated-measures ANOVAs examining gender differences across the six measurement waves.

Factor	Social identity				Civic engagement				Vitality			
	Df num	Df den	F	p	Df num	Df den	F	p	Df num	Df den	F	p
Gender	1	30	2.49	.125	1	23	3.33	.081	1	30	2.07	.160
Time	5	26	1.65	.181	5	19	1.71	.181	5	26	1.24	.319
Gender* Time	5	26	1.15	.362	5	19	.75	.596	5	26	1.41	.254

Time comprised six measurement waves. Gender was included as the between-subjects factor. Heteroscedasticity-consistent standard errors were obtained using the White adjustment.

Overall, these supplementary analyses did not provide evidence of statistically significant gender differences in the average trajectories of social identity, civic engagement, or vitality across the six measurement waves.

### 5.3 Model fit

The longitudinal contemporaneous crossed- effects panel model (**Figure 3**) demonstrated an acceptable fit to the data, indicating that the hypothesized structure adequately represented the observed relationships over time. Specifically, the model yielded a chi-square value of χ²(115), 184.17, *p* <.001, a Comparative Fit Index (CFI) of 0.931, and a Tucker–Lewis Index (TLI) of 0.897. The Root Mean Square Error of Approximation (RMSEA) was 0.053, with a 90% confidence interval ranging from 0.038 to 0.067, and the Standardized Root Mean Square Residual (SRMR) was 0.119. The SRMR value was slightly above the conventional cutoff (.08), a common occurrence in complex multi-wave panel models, likely due to the high number of observed variables and inter-wave correlations (see 64). Overall, these indices fall within the conventional thresholds for acceptable model fit, suggesting that the longitudinal relations among social identity, civic engagement, and vitality were adequately captured by the model.

**Figure 3:**
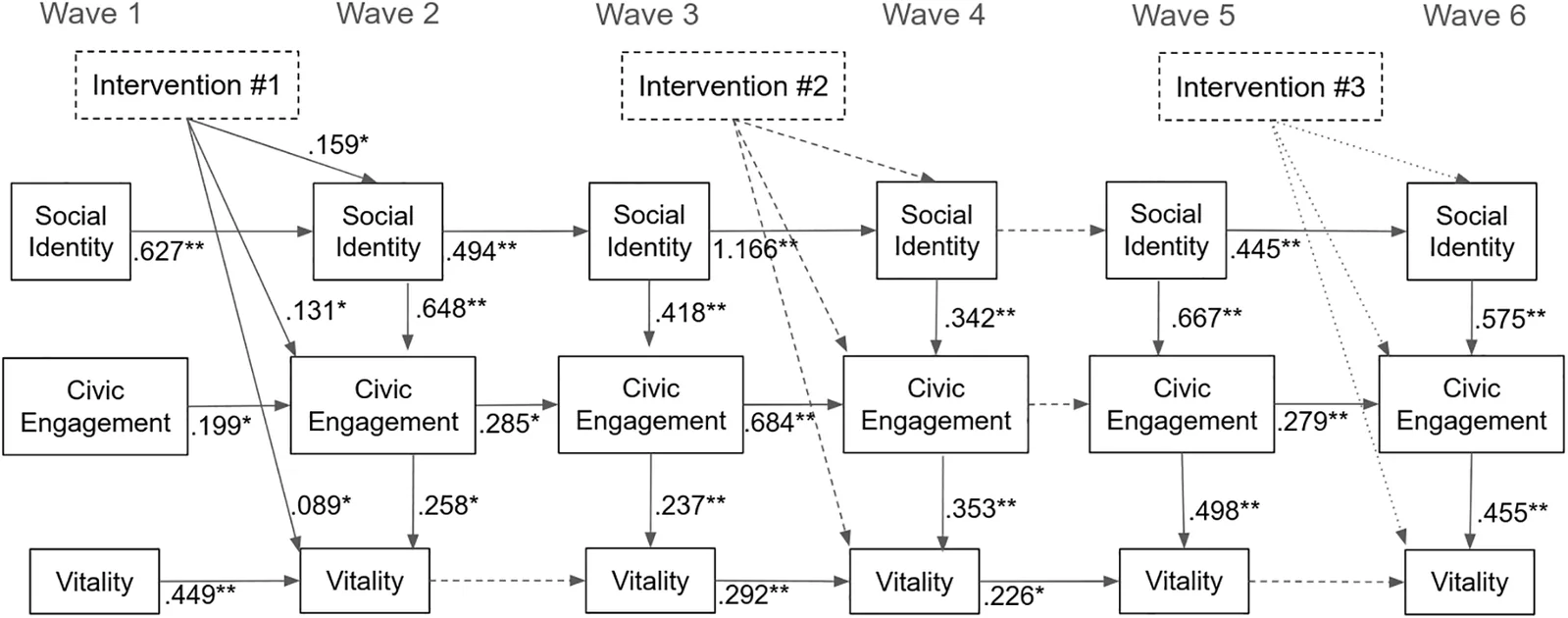
Estimated path model. Non-significant paths are drawn as dashed paths (- - -); Non estimated path for identification issues are drawn as dotted paths (…); Effects of Intervention 1 on variables at Wave 3 are not significant and are not shown to maintain visual clarity; effects of Intervention 2 on variables at Wave 5 are not significant and are not shown to maintain visual clarity; *p <.05; **p <.01.

### 5.4 Autoregressive paths

As shown in **Table 6**, the majority of the standardized autoregressive paths for social identity, civic engagement, and vitality were statistically significant, reflecting moderate to strong temporal stability across measurement waves. Notably, some coefficients—particularly for civic engagement and vitality in the mid phases—were weaker or non-significant, suggesting fluctuations in stability over time.

### 5.5 Longitudinal effects

The longitudinal path estimates (see **Table 6**) provided support for the hypothesized temporal ordering of the constructs.

The intervention group (as opposed to the control group) showed a positive increase in early levels of both social identity (T1→T2: *β* = .159, *p* = .046) and civic engagement (T1→T2: *β* = .131, *p* = .011), indicating that participation in the intervention facilitated increases in these social-psychological resources during the initial phase of the program. However, the differential effect of the intervention group did not emerge as significant on successive waves.

Social identity significantly and positively predicted civic engagement across multiple time lags. For example, higher levels of social identity at T2 were associated with increased civic engagement at T2 (β = 0.648, *p* < .001), and similar effects were observed at T5 (β = 0.667, *p* < .001) and at T6 (β = 0.575, *p* < .001). In turn, civic engagement significantly predicted subsequent levels of vitality at later time points. Specifically, greater civic engagement at T2 predicted higher vitality at T2 (β = 0.258, *p* = .015), at T5 predicted vitality at T5 (β = 0.498, *p* < .001), and at T6 predicted T6 vitality (β = 0.455, *p* < .001).

### 5.6 Indirect effects

Monte Carlo simulations revealed that only one indirect pathway reached significance (**Table 7**). Specifically, the intervention at T1 has an indirect effect on vitality at T2 through the social identity and civic engagement at T2 (standardized indirect effect estimate, 0.027, 95% CI [0.000, 0.069]). No other indirect effects across subsequent time lags were statistically significant. This finding suggests that the pathway from social identity to civic engagement acts as a meaningful mechanism linking early identity processes to later vitality during the initial intervention phase. These longitudinal and indirect associations are summarized in **Tables 6** and **7**, respectively.

**Table 6:** Standardized longitudinal and indirect effects among social identity, civic engagement, and vitality.

					95% C.I.	
Dependent variable	Predictor	β	se	p	LL	UL
Social identity (T2)						
	Social Identity (T1)	.627	.061	.000	.508	.746
	R	.159	.078	.040	.007	.312
Civic engagement (T2)						
	Civic Engagement (T1)	.199	.079	.012	.044	.355
	Social Identity (T2)	.648	.064	.000	.522	.773
	R	.131	.053	.013	.028	.234
Vitality (T2)						
	Vitality (T1)	.449	.075	.000	.303	.596
	Civic Engagement (T2)	.258	.100	.010	.062	.455
	R	.089	.035	.011	.020	.157
Social identity (T3)						
	Social Identity (T2)	.494	.162	.002	.177	.810
	R	-.016	.085	.854	-.182	.150
Civic engagement (T3)						
	Civic Engagement (T2)	.285	.117	.015	.055	.515
	Social Identity (T3)	.418	.113	.000	.197	.638
	R	-.055	.082	.504	-.217	.106
Vitality (T3)						
	Vitality (T2)	.142	.169	.401	-.189	.472
	Civic Engagement (T3)	.237	.073	.001	.094	.380
	R	.079	.095	.403	-.107	.265
Social identity (T4)						
	Social Identity (T3)	1.166	.189	.000	.795	1.537
	R	.031	.095	.743	-.155	.217
Civic engagement (T4)						
	Civic Engagement (T3)	.684	.189	.000	.314	1.054
	Social Identity (T4)	.342	.107	.001	.133	.551
	R	.053	.092	.570	-.129	.234
Vitality (T4)						
	Vitality (T3)	.292	.110	.008	.076	.508
	Civic Engagement (T4)	.353	.095	.000	.168	.538
	R	.062	.086	.471	-.107	.232
Social identity (T5)						
	Social Identity (T4)	.438	.254	.084	-.060	.935
	R	.160	.092	.082	-.020	.340
Civic engagement (T5)						
	Civic Engagement (T4)	.015	.091	.872	-.164	.193
	Social Identity (T5)	.667	.065	.000	.539	.795
	R	.073	.077	.341	-.077	.224
Vitality (T5)						
	Vitality (T4)	.226	.093	.015	.044	.409
	Civic Engagement (T5)	.498	.063	.000	.375	.621
	R	-.043	.083	.607	-.206	.121
Social identity (T6)						
	Social Identity (T5)	.445	.102	.000	.245	.645
Civic engagement (T6)						
	Civic Engagement (T5)	.279	.106	.008	.071	.487
	Social Identity (T6)	.575	.089	.000	.401	.749
Vitality (T6)						
	Vitality (T5)	.137	.072	.058	-.004	.278
	Civic Engagement (T6)	.455	.094	.000	.271	.638

R, Control vs Intervention.

**Table 7:** Completely standardized indirect effect estimates and corresponding Monte Carlo 95 C.I.

Indirect effects		Monte Carlo 95% C.I.	
	β	LL	*UL*
Intervention (T1) → Social Identity (T2) → Civic Engagement (T2) → Vitality (T2)	.027	.0001	*.069*
intervention (T3) → Social Identity (T4) → Civic Engagement (T4) → Vitality (T4)	–.002	–.023	*.015*

Indirect effects were tested using Monte Carlo simulations (50,000 replications). LL, Lower Limit; UL, Upper Limit.

## 6 Discussion

### 6.1 General findings

This study provides robust longitudinal evidence that social identity functions as a key antecedent of civic engagement, which subsequently fosters vitality among secondary school students. By tracking developmental changes across six time points within one academic year, the findings offer strong temporal support for a sequential pathway in which adolescents’ sense of belonging to valued social groups, including more inclusive and superordinate forms of identification, motivates them to participate more actively in civic life, which in turn promotes their psychological energy and well-being. These results align closely with the propositions of social identity theory (4), which emphasizes the motivational force of group identification, and self-determination theory (14), which highlights how meaningful social connections and autonomy-supportive contexts enhance intrinsic motivation and vitality. Importantly, the construct of social identity considered in this study reflects a more inclusive and superordinate form of identification, encompassing a sense of belonging to humanity and the natural world, rather than exclusively to proximal groups such as school or peer networks.

The school-based outdoor nature education activities played a central role in shaping these developmental trajectories. Students in the intervention group exhibited elevated levels of both social identity and civic engagement during the early phases of the program. This early pattern is consistent with recent reviews indicating that school-led nature interventions and outdoor education programs may enhance positive affect and social relationships, and that environmental education programs can support civic outcomes when they incorporate sustained, participatory, and action-oriented components (8, 26). It is also compatible with recent Italian intervention evidence showing that school-based nature-related programs can improve selected dimensions of well-being and autonomy, while effects on other constructs may remain limited or inconsistent (23, 45). Taken together, this evidence suggests that nature-based education should not be regarded as a uniformly effective intervention. Its outcomes are likely to depend on the degree to which activities create meaningful social roles, continuity across time, direct contact with nature, and opportunities for agency and collaborative action. This suggests that structured outdoor learning environments—characterized by shared group experiences, environmental exploration, and community-based projects—may promote social-psychological growth (65). Such contexts likely provided adolescents with opportunities to strengthen shared and more inclusive forms of social identification, participate in meaningful collective action, and experience the psychological benefits of agency and belonging (66). Notably, these effects were not transient but were detectable across multiple measurement waves, indicating that identity-building and civic participation are mutually reinforcing processes that can be activated and sustained through well-designed nature-based educational experiences.

The variability of effects across waves should also be considered in light of the heterogeneity documented in the recent literature. Systematic reviews indicate that outdoor and nature-based interventions differ markedly in frequency and duration, educational objectives, pedagogical approach, degree of nature contact, characteristics of supervision, and degree of student-centered participation (25, 26, 67–69). In the present study, the three schools implemented different combinations of excursions, biodiversity activities, botanical laboratories, outdoor reading, and group reflection. Consequently, the common intervention label should not be interpreted as implying identical exposure. Rather, the findings represent the average effect of a family of school-based outdoor nature education programs implemented in distinct geographical and organizational contexts.

The longitudinal measurement-invariance analyses provide additional support for the interpretation of the main findings. Social identity, civic engagement and vitality achieved configural, metric, and scalar invariance across the six waves, indicating that the same underlying constructs were assessed using a sufficiently stable measurement framework over time. Accordingly, the observed longitudinal associations are unlikely to be attributable merely to substantial changes in factor structure, indicator meaning, or response-scale use. Measurement invariance does not imply that the constructs themselves remained unchanged; rather, it indicates that change was assessed using the same unit of measurement across measurement occasions.

At the same time, strict invariance was not fully supported for social identity and civic engagement, whereas vitality achieved strict invariance. This pattern suggests that the core latent meaning of social identity and civic engagement remained stable, but that the amount of indicator-specific variance or measurement error changed across waves. Such residual variability may be plausible for socially embedded constructs that are sensitive to changing peer relations, school contexts and the specific content of the outdoor activities. Nevertheless, because invariance was tested in the pooled sample and civic engagement and vitality were represented through item parcels, the present analyses cannot identify the specific indicators, groups or intervention phases responsible for this variability.

Supplementary analyses did not detect significant gender differences in the mean trajectories of the three constructs. These results suggest that the overall patterns were not markedly different between boy and girl students within the analyzed sample. However, the analyses examined mean-level trajectories rather than gender moderation of the intervention effects or of the structural paths. Therefore, the absence of statistically significant differences should not be interpreted as evidence of complete gender equivalence or as a formal statistical control of gender in the longitudinal model. Previous research also suggests that gender-related differences in adolescent civic engagement may vary according to the specific civic competence or behavioral dimension considered (70).

Within this sufficiently stable measurement framework, the structural results can be interpreted as reflecting longitudinal relationships among the constructs rather than substantial changes in what the measures represented across occasions.

#### 6.1.1 Contemporary and lagged paths from social identity to civic engagement

A closer examination of the longitudinal paths reveals that social identity at earlier time points consistently predicted higher levels of civic engagement at subsequent waves, even though through the autoregressive paths. For example, students who reported stronger identification with valued social groups, particularly at more inclusive levels (e.g., humanity and the natural world), at T2 showed significantly higher engagement at T3, and similar patterns emerged across later waves (e.g., T5 and T6). These lagged relationships indicate that when adolescents perceive a stronger sense of belonging to broader social and ecological communities they are more likely to translate this psychological connection into active civic behaviors over time, consistent with previous studies (71).

The mechanisms underlying these effects can be interpreted through both motivational and developmental lenses. Motivationally, social identification fosters internalization of group norms and goals, providing adolescents with a sense of purpose and responsibility that encourages participation in collective endeavors (20). Developmentally, early adolescence (ages 12–14) is a critical period for identity consolidation and exploration of social roles; thus, a sense of belonging to broader social groups during this stage lays the groundwork for subsequent civic involvement (72).

The structure of the outdoor nature education program helps to explain the timing of these effects. Each intervention’s wave (T1–T2, T3–T4, T5–T6) created intensive periods of shared outdoor activities—such as environmental projects, excursions, and teamwork—that likely strengthened students’ group identity, potentially fostering not only group cohesion but also a more expansive sense of identification with broader social and ecological systems. These identity gains, in turn, translated into increased civic engagement in the following measurement wave, demonstrating a clear lagged effect. Interestingly, the strongest identity-to-engagement effects were observed in the early phases of the study, immediately following the first intervention period. This suggests that the initial exposure to structured outdoor experiences may have had a particularly powerful role in activating adolescents’ civic motivation. Over time, these effects remained significant but showed some variability, likely reflecting differences in program content across schools and potential fluctuations in students’ developmental trajectories.

#### 6.1.2 Contemporary and lagged paths from civic engagement to vitality

The second major set of longitudinal paths concerns the predictive role of civic engagement for subsequent vitality. Considering the combination of contemporary and autoregressive paths across several lags, adolescents who reported higher levels of engagement at earlier time points subsequently exhibited higher vitality, indicating that the perception and feeling of an active involvement in civic and community-oriented activities can serve as a meaningful source of psychological energy and well-being. These results are consistent with the broaden-and-build perspective (73) on positive youth development, which posits that sustained participation in prosocial activities expands adolescents’ personal and social resources, thereby promoting flourishing.

In particular, combining autoregressive with contemporary paths, engagement measured after the intervention waves (e.g., T2 and T3) was a significant predictor of vitality at subsequent time points (T3 and T4, respectively). These findings suggest that nature outdoor activities—especially those embedded in collaborative and environmentally meaningful contexts—play a crucial role in energizing adolescents (8). Participation in such activities likely fosters a sense of competence, purpose, and relatedness, which are core components of vitality (15, 44). Moreover, environmental projects and outdoor educational experiences often produce immediate tangible outcomes (e.g., planting trees, improving school gardens), which can enhance feelings of efficacy and pride, further boosting psychological energy (74).

It is worth noting that even though the contemporary effects are always significant across all waves, not all autoregressive paths reached statistical significance, particularly after the mid-phase of the study (e.g., T4–T5). This pattern can be partly explained by the cyclical structure of the outdoor education activities. After the second intensive intervention period, social identity and engagement levels may have undergone a change that led to weaker predictive associations. This does not necessarily indicate a lack of relationship but rather reflects the dynamic and time-sensitive nature of program-induced changes. Such temporal fluctuations underscore the importance of intervention timing in longitudinal models: effects tend to peak immediately following structured activities and may attenuate in between waves if not continuously reinforced.

Taken together, these findings highlight the developmental significance of social identity processes during adolescence and the pivotal role that school-based outdoor nature education programs can play in activating and sustaining these pathways. Early boosts in social identification catalyze subsequent civic engagement, which then contributes to enhanced vitality and well-being. These processes appear strongest during initial intervention phases, suggesting that early engagement is particularly critical for setting positive developmental trajectories. Over time, these dynamics can create positive feedback loops, whereby strengthened identities foster engagement, which in turn reinforces vitality and potentially leads to further identification with valued groups.

### 6.2 Theoretical and practical implications

From a theoretical perspective, these findings contribute to a more integrated and dynamic understanding of how social identity and civic engagement jointly shape adolescents’ well-being over time. Importantly, the form of social identity examined in this study reflects a superordinate level of identification, extending beyond proximal group memberships to encompass a broader sense of belonging to humanity and the natural world. This suggests that identity processes relevant to adolescent development may operate not only at local group levels but also at more inclusive and global levels, particularly in contexts such as nature-based outdoor education. This sequential process suggests that identification with meaningful social groups does not merely influence outward social behaviors but also fosters internal psychological resources and energy. Moreover, the longitudinal design allows for the examination of how these processes developmentally unfold, highlighting that early boosts in identity and engagement can initiate positive feedback loops then sustaining well-being across time. The integration of identity and motivational frameworks also extends current theories by demonstrating how environmental and social contexts—such as outdoor civic education—can serve as catalysts that activate these developmental mechanisms.

From a practical perspective, the results provide valuable insights for educators, practitioners, and policymakers seeking to design impactful school-based programs. Interventions that intentionally cultivate students’ sense of belonging and provide authentic opportunities for civic action can yield lasting benefits for both personal development and collective outcomes. Outdoor and community-based learning contexts, in particular, appear to be especially effective in combining identity-building, engagement, and well-being promotion. Investing in such initiatives early in adolescence is crucial, as initial experiences can set developmental trajectories that are self-reinforcing over time. Furthermore, these findings support the idea that schools are not merely sites of academic instruction but also powerful developmental ecosystems that shape students’ civic identities and psychological flourishing. Embedding civic and identity-focused components into the broader curriculum can help create inclusive educational environments that foster both democratic participation and individual well-being in the long term.

### 6.3 Limitations and future research directions

Despite these contributions, several limitations should be acknowledged. First, the study relied exclusively on self-report measures, which may be subject to social desirability and common method biases. Although all scales were well-validated and translation procedures ensured linguistic equivalence, future research could incorporate multi-informant reports, objective behavioral indicators (e.g., documented civic activities), or physiological measures of vitality to strengthen the validity and robustness of the findings.

Second, the intervention design—implemented between three pairs of measurement points (T1–T2, T3–T4, and T5–T6)—may have introduced complexities in interpreting temporal change. While this repeated-intervention structure reflects real-world educational practice, it complicates efforts to disentangle short-term from cumulative intervention effects. For example, improvements observed in later waves may reflect the additive impact of multiple exposures rather than the sustained effect of earlier phases. Future studies could address this issue by introducing control groups for each intervention wave, employing staggered intervention designs, or applying latent growth curve modeling to more accurately capture developmental trajectories.

Third, because students were drawn from three different schools, the content and intensity of the outdoor education activities varied across sites, reflecting differences in environmental resources, partnerships, and implementation fidelity. This variability may have introduced heterogeneity in intervention effects, potentially attenuating or inflating certain pathways. Future research should consider systematically assessing implementation differences across schools, for example by incorporating fidelity checklists or multilevel modeling to account for school-level variance. Furthermore, because the participating schools were in different geographical contexts, part of the observed variability may also reflect contextual characteristics that were not directly measured in the present study.

Fourth, another limitation concerns the absence of direct measures of individual and contextual characteristics that may influence adolescents’ social-psychological development. Although age and gender were recorded, no specific information was collected regarding participants’ socioeconomic status, family background, cultural background, or physical and mental health. Previous research suggests that these factors may shape social identity formation, civic engagement, and psychological well-being. Consequently, the longitudinal associations should be interpreted within the broader educational and social contexts in which the interventions were implemented. Future studies should include these variables to better define intervention effects.

Fifth, although missing data were handled appropriately through modern estimation methods, attrition and partial non-response across six waves may still have reduced statistical power or biased parameter estimates. In addition, the overall model fit indices were acceptable but not optimal, which may be partly due to the complexity of the six-wave longitudinal structure and the presence of missing data. A larger number of measurement occasions increases model complexity, making it more difficult to achieve excellent fit indices, especially when some paths are weak or non-significant. Future studies should aim to minimize missing data through improved follow-up procedures, consider simplifying model structures where theoretically justified, and apply sensitivity analyses to evaluate the robustness of longitudinal effects.

Finally, the sample consisted of Italian secondary school students, which may limit the generalizability of the findings to other cultural and institutional contexts. Cross-cultural longitudinal studies could examine whether these mechanisms operate similarly across diverse educational systems and sociocultural settings. Moreover, while the present study spanned one academic year, longer-term follow-ups would help assess the durability of observed effects and detect potential delayed or emergent developmental pathways.

## 7 Conclusion

This study provides evidence that fostering adolescents’ identity through outdoor nature-based educational experiences can support civic engagement and psychological well-being. Beyond demonstrating specific intervention effects, the findings highlight the broader developmental role of identity-building processes and the potential of experiential learning environments to enhance social and motivational outcomes.

Importantly, school-based outdoor programs offer unique contexts in which adolescents can explore collective identities, collaborate with peers, and engage in meaningful community activities. By leveraging these opportunities, educators can design interventions that promote both personal flourishing and active participation in civic and environmental initiatives.

These results also suggest that the relationships among identity, engagement, and vitality are dynamic and context-dependent, underscoring the need for flexible and responsive program design. Future research could further examine contextual moderators, school-level variability, and longer-term trajectories to refine our understanding of how nature-based interventions shape adolescent development.

Overall, this study reinforces the value of outdoor educational experiences not only as tools for environmental and civic education but also as developmental platforms that nurture social-psychological well-being during a critical period of life.

## Data Availability

The raw data supporting the conclusions of this article will be made available by the authors, without undue reservation.

## Supplementary material

Supplementary File 1 Survey materials used in the study, including the informed consent forms, Social Identity, Civic Engagement, and Subjective Vitality measures, and **Supplementary Table S1** reporting the effects of attrition (drop-out) on the main outcome variables.
