# Supplementary material for: Outdoor school-based nature education programs improve social life and vitality: evidence from a six-wave longitudinal study on Italian secondary school students
Source: Front Psychiatry. 2026 Sep 23;17:1913900. doi: 10.3389/fpsyt.2026.1913900 (PMC13642832; doi:10.3389/fpsyt.2026.1913900)
Supplement: Supplementary file 1 [file SupplementaryFile1.docx]

**Appendix**

The Survey for the Study

Per favore, leggi attentamente.

**CONSENSO INFORMATO**

Titolo della ricerca: Gli effetti psicologico-sociali dell’Outdoor Education su studenti e studentesse

Responsabile scientifico: Prof. Marino Bonaiuto (marino.bonaiuto@uniroma1.it) Prima di decidere liberamente se vuoi partecipare alla ricerca, LEGGI ATTENTAMENTE questo consenso informato e poni tutte le domande che ritieni opportune al fine di chiarire gli eventuali dubbi sugli scopi, le modalità di esecuzione della ricerca e i possibili inconvenienti connessi. Ti preghiamo di ricordare che questo è un progetto di ricerca e che la tua partecipazione è completamente volontaria.

**OBIETTIVO DELLO STUDIO:** Il questionario che ti sottoponiamo è finalizzato a valutare come l'ambiente scolastico esterno influisca sulla cognizione, sulle emozioni, sulle relazioni sociali e sul benessere degli studenti e delle studentesse. I risultati saranno analizzati e pubblicati in forma anonima e aggregata.

**PROCEDURE DELLO STUDIO:** Invitiamo i/le partecipanti alla ricerca a rispondere alle domande secondo le modalità di volta in volta proposte. In molti casi si tratta di dichiarare il proprio grado di accordo con affermazioni riguardanti atteggiamenti, norme, credenze e comportamenti legati alla salvaguardia dell’ambiente. Verranno richieste, inoltre, alcune informazioni personali riguardanti genere, età e luogo di residenza.

**TRATTAMENTO DEI DATI: I dati saranno trattati unicamente in forma aggregata e potranno essere utilizzati solamente per finalità scientifiche o divulgative (ad es. tesi di laurea, articoli scientifici, relazioni in congressi, etc.).**

**RISCHI, DISAGI ED EFFETTI COLLATERALI:** La partecipazione alla ricerca NON comporta rischi o disagi fisici e/o emotivi rilevanti per la salute. Ti ricordiamo che potrai sospendere la compilazione e ritirarti dalla ricerca IN QUALSIASI MOMENTO senza alcun obbligo di motivare la tua decisione.

Il tuo consenso attesta che:

1. Hai letto le informazioni sullo scopo e le procedure della ricerca e hai ricevuto tutte le informazioni relative alla procedura;

2. Hai accettato di partecipare allo studio per il quale si sta richiedendo consenso alla protezione dei dati.

Dopo aver letto la parte informativa sugli scopi e le procedure di questa ricerca dichiaro di:

- Acconsentire alla partecipazione alla ricerca
- NON acconsentire alla partecipazione alla ricerca

**INFORMED CONSENT**

**Title of the research:** The psychosocial effects of Outdoor Education on students

**Scientific supervisor: Prof. Marino Bonaiuto (marino.bonaiuto@uniroma1.it)**

Before deciding freely whether you wish to take part in this study, PLEASE READ THIS INFORMED CONSENT CAREFULLY and feel free to ask any questions you may have in order to clarify any doubts about the aims, procedures, or possible inconveniences related to the research. Please remember that this is a research project and that your participation is entirely voluntary.

**STUDY OBJECTIVE:** The purpose of this questionnaire is to assess how the outdoor school environment influences students’ cognition, emotions, social relationships, and overall wellbeing. The results will be analysed and published in anonymous and aggregated form.

**STUDY PROCEDURES:** Participants are invited to respond to the questions according to the instructions provided. In many cases, you will be asked to indicate your level of agreement with statements concerning attitudes, norms, beliefs, and behaviours related to environmental protection. You will also be asked to provide some personal information such as gender and age.

**DATA PROCESSING:** All data will be processed exclusively in aggregated form and may be used only for scientific or dissemination purposes (e.g., degree theses, scientific articles, conference presentations, etc.).

**RISKS, DISCOMFORTS, AND SIDE EFFECTS:** Participation in this research does NOT involve any physical and/or emotional risks or discomforts relevant to your health. Please note that you may interrupt the questionnaire and withdraw from the study AT ANY TIME without being required to provide any explanation.

Your consent confirms that:

1. You have read the information about the purpose and procedures of the study and have received all relevant details regarding the procedure;
2. You agree to take part in the study for which consent to data processing is being requested.

After reading the information about the aims and procedures of this study, I declare that I:

☐ **Consent** to participate in the research

☐ **Do NOT consent** to participate in the research

Genere:

- Maschio
- Femmina
- Genere non-binario / Terzo genere
- Preferisco non dirlo

**Gender:**

- Boys
- Girls
- Non-binary / Third gender
- Prefer not to say

Età
________

Age

_________

Social Identity

- I see myself as a global citizen.
- I feel connected with the whole people living in the earth.
- I feel as a part of the earth population.

*Per favore esprimi il tuo grado di accordo/disaccordo con le seguenti affermazioni tenendo conto della scala di riposta:*

*(1 = completamente in disaccordo; 7 = completamente d’accordo)*

- *Mi considero cittadino del mondo*
- *Sento di avere legami con tutte le persone che vivono nel mondo*
- *Mi vedo come parte della popolazione terrestre.*

Civic Engagement

- I believe I should make a difference in my school community.
- I am committed to serve in my school community.
- I believe that all citizens have a responsibility to their community.
- When working with others, I make positive changes in the school community.
- I help members of my school community.
- I stay informed of events in my school community.

*In riferimento al quartiere nel quale vai a scuola, ti chiediamo di indicare il tuo grado di accordo o disaccordo con le seguenti affermazioni riportate qui sotto, tenendo presente la scala di risposta (1=fortemente in disaccordo; 5=fortemente d'accordo)*

- *Credo di dover fare la differenza nella mia comunità scolastica.*
- *Mi impegno al servizio della mia comunità scolastica.*
- *Credo che tutti gli/le studenti/esse abbiano una responsabilità nei confronti della propria comunità scolastica.*
- *Quando collaboro con altri, apporto cambiamenti positivi nella comunità scolastica.*
- *Aiuto i membri della mia comunità scolastica.*
- *Mi tengo informato sugli eventi della mia comunità scolastica*

Vitality

Please respond to each of the following statements by indicating the degree to which the statement is true for you in general in your life. (1=not at all true; 4=somewhat true; 7=very true)

- I feel alive and vital.
- I don’t feel very energetic.
- I have energy and spirit.
- I look forward to each new day.
- I nearly always feel alert and awake.
- I feel energized.

*Per favore rispondi a ciascuna delle seguenti affermazioni indicando il grado in cui essa è vera per te in generale nella tua vita. (1 = per niente vera; 4 = abbastanza vera; 7 = completamente vera)*

- Mi sento vivo e vitale.
- Non mi sento molto energico.
- Ho energia e spirito.
- Non vedo l’ora che arrivi ogni nuovo giorno.
- Mi sento quasi sempre vigile e sveglio.
- Mi sento pieno di energia.

**Supplementary Materials**

**Table S1** - Effects of Attrition (drop-out) on main outcome variables

|  |  | Df num | Df den | F | p |
| --- | --- | --- | --- | --- | --- |
| Social Identity |  |  |  |  |  |
|  | Dropout at T2 | 1 | 127 | .026 | .871 |
|  | Dropout at T3 | 1 | 127 | .183 | .669 |
|  | Dropout at T4 | 1 | 127 | .073 | .787 |
|  | Dropout at T5 | 1 | 127 | 2.310 | .131 |
|  | Dropout at T6 | 1 | 127 | 1.410 | .237 |
| Civic Engagement |  |  |  |  |  |
|  | Dropout at T2 | 1 | 127 | 1.969 | .163 |
|  | Dropout at T3 | 1 | 127 | .532 | .467 |
|  | Dropout at T4 | 1 | 127 | .030 | .863 |
|  | Dropout at T5 | 1 | 127 | .070 | .792 |
|  | Dropout at T6 | 1 | 127 | .094 | .759 |
|  |  |  |  |  |  |
| Vitality | Dropout at T2 | 1 | 127 | 5.950 | .016 |
|  | Dropout at T3 | 1 | 127 | 3.000 | .086 |
|  | Dropout at T4 | 1 | 127 | .755 | .387 |
|  | Dropout at T5 | 1 | 127 | .846 | .359 |
|  | Dropout at T6 | 1 | 127 | .532 | .467 |
